# MetaBinG: Using GPUs to Accelerate Metagenomic Sequence Classification

**DOI:** 10.1371/journal.pone.0025353

**Published:** 2011-11-23

**Authors:** Peng Jia, Liming Xuan, Lei Liu, Chaochun Wei

**Affiliations:** 1 Department of Bioinformatics and Biostatistics, School of Life Sciences and Biotechnology, Shanghai Jiao Tong University, Shanghai, China; 2 Key Laboratory of Systems Biology, Shanghai Institutes for Biological Sciences, Chinese Academy of Sciences, Shanghai, China; 3 Graduate School of the Chinese Academy of Sciences, Shanghai, China; 4 Shanghai Center for Bioinformation Technology, Shanghai, China; 5 Department of Biochemistry and Molecular Biology, School of Bioengineering, East China University of Science and Technology, Shanghai, China; J. Craig Venter Institute, United States of America

## Abstract

Metagenomic sequence classification is a procedure to assign sequences to their source genomes. It is one of the important steps for metagenomic sequence data analysis. Although many methods exist, classification of high-throughput metagenomic sequence data in a limited time is still a challenge. We present here an ultra-fast metagenomic sequence classification system (MetaBinG) using graphic processing units (GPUs). The accuracy of MetaBinG is comparable to the best existing systems and it can classify a million of 454 reads within five minutes, which is more than 2 orders of magnitude faster than existing systems. MetaBinG is publicly available at http://cbb.sjtu.edu.cn/~ccwei/pub/software/MetaBinG/MetaBinG.php.

## Introduction

The culture-independent metagenomics methods try to sequence all genetic materials recovered directly from an environment. It has the potential to provide a global view of a microbial community [1]. However, one of the challenging tasks is to assign these raw reads or assembled contigs into classes according to the evolutionary distances among their source genomes. This process is called metagenomic sequence classification.

There are two major types of computational methods for metagenomic sequence classification: alignment-based and composition-based. Alignment-based methods can determine that a sequence is from an organism only if the source genome or a genome with similar sequence has been sequenced. When the source genome is fully sequenced, alignment-based methods are accurate in general. However, it is difficult for alignment-based methods to do classification when the sequences of the source genomes or closely-related genomes are not available. Unfortunately, this is the case for many metagenomes. It is a significant limitation for alignment-based methods. Composition-based methods, on the other hand, are less accurate but are able to assign every read to a source bin, which can be one or more species, genera or other taxonomy ranks.

Advantages of the next-generation sequencing (NGS) technologies such as the high throughput and low cost make them more and more attractive for metagenome sequencing. Among NGS platforms, the 454 sequencers provide the longest reads (up to 400 bps in average), and they can generate more than half a million reads in just one run [2]. However, NGS technologies make classification more challenging by providing a large amount of shorter reads than a traditional sequencing platform does. These fast growing numbers of metagenomic sequences from NGS platforms put efficient and reliable classification systems in high demand.

There are many existing metagenomic sequence classification systems, such as Phymm and PhymmBL [3]. Phymm uses interpolated Markov models (IMMs) to classify short reads, and has obtained pretty good sensitivity and specificity on its own test dataset. However, the computational cost of Phymm is very expensive and it can be a problem when the size of a dataset to be classified is huge. For example, it may take Phymm 100 hours or more to classify a single run of 454 sequencing data (see Results part). PhmmBL added alignment-based method to Phymm, and achieved better accuracy. PhymmBL is about 50% slower than Phymm (data not shown). Recent updated version of PhymmBL [4] can run multiple jobs simultaneously in a multi-processor computer. PhymmBL can also run on multiple machines. But this parallelization of PhymmBL requires extra splitting and merging steps for each list of input reads. For other similar systems with webservers, such as CAMERA [5], MG-RAST [6], [7], the time to classify a run of 454 sequencing data varies from hours to weeks [8]. This can be a serious problem when the sample size increases.

Graphic processing units (GPUs) were originally designed to accelerate graphic display only. In the past few years, GPUs have evolved to GPGPUs (general purpose GPUs), which can do general purpose scientific and engineering computing. In many cases, programs implemented on GPUs can run significantly faster than on multi-core CPU-based systems since a GPU may have hundreds of cores. With the success of a parallel programming model called CUDA for GPUs, programming on GPUs for general scientific computing becomes much easier than before [9]. Therefore, using GPUs is becoming very attractive for researchers who need to boost the performance of their applications in a wide range of scientific areas, including bioinformatics [10]. However, it is not straightforward to apply GPUs to a new research area. A GPU-based version of BLAST has been developed, and it is nearly four times faster than the CPU-based version [11].

In metagenomics, “metagenomic sequence classification” is sometimes distinguished from “metagenomic sequence binning, ” which refers to the grouping of a dataset into subgroups but the subgroups remain unlabeled [3]. Obviously, a metagenomic sequence classification system can also be used as a binning system by treating all distinct subgroups without considering their names or labels.

In this paper, we present a fast metagenomic sequence classification system (MetaBinG) using the power of GPUs. MetaBinG is able to classify accurately a single run of NGS-based metagenome shotgun sequence data in minutes instead of hours or days in a single desktop workstation.

## Results and Discussion

In order to compare the performance of MetaBinG and Phymm, 1212 fully sequenced bacterial genomes were downloaded from the NCBI FTP site (ftp://ftp.ncbi.nih.gov/genomes/Bacteria/) on 14 December 2010. With NCBI taxonomy information, 390 genomes were removed to guarantee that every genus has at least two genomes. The remaining 822 genomes belong to 133 genera (534 species). The species in each genus were assigned to training and test groups. All genomes of one species were assigned into a same set, either the training set or the test set. In the end, we generated a training set of 468 genomes (288 species, 133 genera) and a test set of 354 genomes (246 species, 133 genera) (The complete list of training and test genomes is available as Table S1). This simulated the situation that the source organisms of short sequences were not present in the reference database. At least at species level, there was no overlap between the training and test sets. Ten different sequence lengths were tested. For each sequence length, 10 sequences were randomly sampled from every chromosome or plasmids of the 354 test genomes. Therefore, there were 6,640 reads for each sequence length. Then the test datasets were classified using MetaBinG trained from the 468 training genomes with K = 5 (We observed that 5th-order Markov model was enough to get accurate results).

Although better results could be achieved with higher order of Markov models, there may be not sufficient data to train the models when K increases. For example, in a 4-million-bp genome, 4^K^ is larger than 4 million when K is bigger than 11. Therefore, there must be insufficient training data for models if K is bigger than 11. In addition, there will be 4 times more computing time when K increases one since the complexity of MetaBinG is 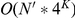, where *N′* is the number of genomes used for training. Therefore, the best result may be obtained by a relatively low order of Markov model (Table 1). The speed of classification is also an important aspect. Considering all these issues, we finally chose 5^th^-order Markov models for metagenomic sequence classification.

**Table 1:** Impact of the order (K) of Markov models in MetaBinG.

Sequence Length (bps)	K = 3	K = 4	K = 5	K = 6	K = 7
100	1/47.5	2/49.6	4/50.6	15/50.6	**55/50.7**
200	2/56.7	2/58.9	5/60.8	**16/61.7**	55/61.6
300	2/62.2	3/64.9	**6/67.7**	16/67.6	57/66.9
400	3/66.3	4/69.7	**6/71.6**	17/71.6	57/71.4
500	3/69.2	4/72.7	**8/74.5**	18/74.5	59/73.3
600	4/71.9	5/75.3	8/77.2	**19/78.0**	59/74.9
700	5/74.5	6/77.8	9/79.2	**20/79.3**	61/76.9
800	6/75.4	7/78.4	**10/80.3**	22/79.9	62/77.2
900	8/76.6	9/79.2	**12/80.8**	22/80.6	63/78.4
1000	8/78.5	10/81.5	13/82.4	**25/82.7**	65/78.9

The impact of the order of Markov models (K) in MetaBinG has been tested. The K values various from 3 to 7. The sequence data sets are the same as in Table 2. Ten different sequence lengths from 100 bps to 1000 bps have been used for testing. Each sequence length contains 6,640 sequences. Each column is for a K value, which is the order of a Markov model. The total computing time (in seconds) and accuracy was measured as in Table 2. Each cell contains the total computing time and the accuracy separated by a “/”.

For each sequence length, the best performance is in a bold font. K is set to 5 by default in MetaBinG.

Both MetaBinG and Phymm V3.2 perform well at high ranks (Table 2). Although the accuracy of MetaBinG is lower than Phymm in most cases, the differences between MetaBinG and Phymm decrease when the read length increases. MetaBinG performs classification at all taxonomy levels. The accuracy results at the lower ranks (from phylum to genus) were reported in Table S2 and S3 for Phymm and MetaBinG respectively. The results show that the MetaBinG is at least 140-fold faster than Phymm with a comparable or slightly worse accuracy (Table 2). When applied to real data, the high-order Markov models can be pre-computed from all the 1212 genomes for both MetaBinG and Phymm. It took MetaBinG about 3.5 hours to build 5^th^-order Markov Models for 1212 genomes with a single thread version training program, while Phymm spent 30 hours to build IMMs for the 1212 genomes. Unlike Phymm, MetaBinG package contains the pre-built 5^th^-order Markov Models so that users could use MetaBinG directly without any training steps. In addition, we provide an interface to add new training genomes to the pre-built Markov Models.

**Table 2:** Comparison of Phymm and MetaBinG.

Sequence Length (bps)	Phymm		MetaBinG		Speedup
	Accuracy (%)	Time (s)	Accuracy (%)	Time (s)	
100	53.62	573	50.61	4	143
200	64.21	880	60.82	5	176
300	70.71	1262	67.66	6	210
400	73.36	1652	71.56	6	275
500	76.02	1949	74.48	8	244
600	78.47	2330	77.24	8	291
700	79.89	2632	79.21	9	292
800	81.86	3006	80.25	10	301
900	82.40	3403	80.77	12	284
1000	84.18	3795	82.35	13	292

Ten different sequence lengths from 100 bps to 1000 bps have been used for testing. Each sequence length contains 6,640 sequences. The accuracy and total computing time (in seconds) for 6,640 sequences is listed in the table. Accuracy is measured at phylum level. The last column in the table shows the speedup of MetaBinG compared to Phymm. Both Phymm and MetaBinG were tested in the same Linux machine with 2 Intel Xeon E5520 processors (8 cores in total), 16 GB RAM and one NVDIA Tesla C1060 GPU card (240 cores). Default parameters were used for Phymm. The same input sequences and reference databases were used for both MetaBinG and Phymm. The accuracy is defined by dividing the number of correctly predicted sequences by the total number of test sequences since both methods assign every sequence to a source genome. The time measured here included all overhead except the creating of reference databases.

**Figure 1:**
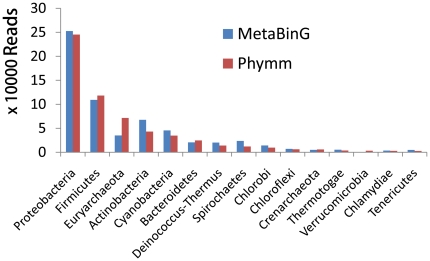
Biogas metagenome recovered by MetaBinG and Phymm. The 616,072 454 reads contained in the biogas metagenome dataset have been classified using MetaBinG and Phymm. The classification accuracy was measured at phylum level. The histogram shows only the top 15 phylum from the metagenomes recovered by Phymm. In general, the results recovered from MetaBinG and Phymm are similar except some small differences in *Euryarchaeota* and *Actinobacteria*. Among the top 15 phyla generated by Phymm, 14 was in the list of top 15 produced by MetaBinG. The relative ranks for these phyla generated by different methods varies at most by a value of two. MetaBinG is almost 1500-fold faster than Phymm.

MetaBinG has been tested on a real dataset, a biogas reactor dataset containing 616,072 454 reads with an average length of 230 bps [12]. Using all the 1212 training genomes, MetaBinG spent 248 seconds and Phymm spent 4 days 5 hours 57 minutes and 56 seconds to classify the biogas reactor dataset, which means MetaBinG is almost 1500-fold faster than Phymm when dealing with real high throughput sequencing data. However, the microbial community recovered by MetaBinG and Phymm is quite similar (Figure 1). In practice, multiple instances of Phymm can be run in a computer with multiple cores. Therefore, the actual speedup may be much lower. For example, the speedup for the biogas dataset analysis will be 188 ( = 1500/8) if 8 instances of Phymm are run simultaneously in one computer with 8 cores. However, it is safe to say that MetaBinG is 2 orders of magnitude faster than Phymm.

It may seem inefficient for sequences with lengths shorter than 4^K^ bases. However, k-th order Markov model provides a uniformed representation (4^k+1^ dimension vectors) for genomes in the reference database, which makes it very simple to parallelize the computing in a GPU. In practice, this computing is done by matrix multiplication. The matrix multiplication functions are from CUBLAS library, which is a Basic Linear Algebra Subprograms (BLAS) library ported to CUDA (Compute Unified Device Architecture) [13]. CUBLAS supports high density of parallelization, and the parallelization is managed by CUDA. In addition, matrix multiplication functions in CUBLAS are optimized for parallel computing in GPUs. Compared to a single threaded CPU version of the same algorithm, the speedup can even be larger than the actual number of cores in GPUs.

In order to check the impact of GPUs on the speed of MetaBinG, MetaBinG and its CPU version were compared. A naive single-threaded version of MetaBinG was implemented without using the BLAS library. K was set to its default value 5. For the same 6,640 input sequences as in Table 2, MetaBinG was about 200 to 500 times faster than its CPU version for sequences with lengths from 100 bps to 1000 bps when all 1212 genomes were included in the reference database (data not shown). In the test for the biogas dataset with more than half million sequences, the speedup could go up to about 600 times. A parallel CPU version of MetaBinG was also implemented using BLAS library (http://www.netlib.org, last updated on Jan. 20^th^, 2011). For the biogas dataset, the GPU version was about 25 times faster than the parallel CPU version (data not shown). Therefore, the speedup of MetaBinG is partially from BLAS library, and partially from GPUs. Meanwhile, the speedup of using GPUs is related to the size of inputs including the number of sequences and the size of reference genome database. The bigger input data sets, the higher speedup it can achieve.

In general, MetaBinG is an ultra-fast metagenomic sequence classification system for high-throughput sequence data. We demonstrated that MetaBinG could provide competitive results for sequences with long lengths in a speed 2 orders of magnitude faster. Due to the progress of sequencing technologies, the throughput gets higher and the reads get longer. The demand for a fast tool to analyze a huge amount of metagenomic sequences is constantly increasing. Therefore, MetaBinG can be a useful tool for the metagenomic classification.

Latest version of PhymmBL can produce a confidence score for an input sequence at each taxonomy rank, which is very convenient for users to assess the reliability of the classification. MetaBinG has not implemented this though it will be a welcome feature to come in the near future.

MetaBinG is publicly available at http://cbb.sjtu.edu.cn/~ccwei/pub/software/MetaBinG/MetaBinG.php. MetaBinG contains a pre-built 5^th^-order Markov Model for each of the existing 1212 genomes, so users do not need to train the models any more. The file size of current version of the full package is about 17 MB. MetaBinG has been tested on 64-bit Linux OS. One CUDA device is needed and it should be installed appropriately before running MetaBinG. In addition, users can add new Markov models (or genomes to the training set) using the addref.pl script in the package. In the near future, MetaBinG may become more accurate with more genomes available. This ultra-fast tool can be useful for a wide range of related research communities.

## Methods

A k^th^-order Markov models is used in MetaBinG. A state in the Markov model is defined as an oligonucleotide of length *k*, and each state connects to 4 other states. The previous state shares *k-1* bases with the next state. Therefore, there are *4^k+1^* transitions in total. A genomic sequence under the k^th^-order Markov model can be viewed as a sequence of state-transitions. The transition probabilities can be calculated for each genome in the training data set according to its Markov model as following:
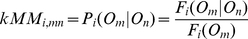
(1)where *O_m_* and *O_n_* are oligonucleotides of length *k*, *P(O_m_ | O_n_)* represents the transition probability from *O_m_* to *O_n_*, *F(O_m_ | O_n_)* represents observed count of transitions from *O_m_* to *O_n_* in a genomic sequence *i* and *F(O_m_)* is the observed count of *O_m_*. A *4^k+1^*diemension vector is created to represent each genome in the training set. In practice, the minus logarithm value of each transition probability is saved.

A short sequence of length *l* can be considered as *l-k* transitions and a score *S_i_*, which represents the distance between the short sequence and a genome *i*, can be computed as following:
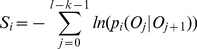
(2)where *O_j_* and *O_j+1_* are two oligonucleotides of length *k*, and *P(O_j_|O_j+1_)* is the transition probability from *O_j_* to *O_j+1_* observed in the *i-th* genome. When the transition from *O_j_* to *O_j+1_* does not exist in the *i-th* genome, the logarithm value of the transition probability will be set to a constant (default is 10). The high-order Markov models can be pre-computed from genomes in the training dataset. For each sequence, a genome in the database with the minimum score is selected as the source genome. At the end, each test sequence will be annotated with the taxonomy information of its source genome.

The algorithm complexity is determined by the number of genomes in the database and the order of Markov Models. It can be defined as follows
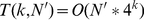
(3)where *k* represents the length of oligonucleotides and *N′* stands for the number of genomes used for training.

In practice, the score *S_i_* in equation (2) is calculated by matrix multiplication. First, the transitions generated from each genome in the reference database are converted into a *4^k+1^*diemension vector. Then, a matrix can be created from all vectors generated from genomes in the reference database. These can be prebuilt. For each short metagenomic sequence, the transitions generated from it are converted into a *4^k+1^*dimension vector as well. Then, the scores are computed by matrix multiplication, which is done by calling the SGEMM() function of CUBLAS library. At the end, the best score is picked and the associated genome is selected as its source genome. These are done by GPUs and the taxonomy information about the source genomes is printed out by CPUs.

**Figure 2:**
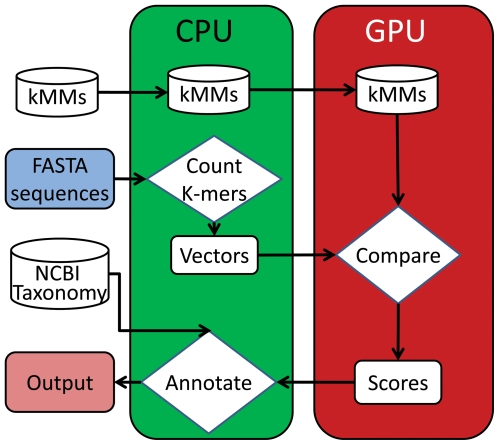
The system design of MetaBinG. First, the pre-built k^th^-order Markov Models (kMMs) are loaded to the GPU memory. Second, a CPU transforms input FASTA sequences into vectors of k-mer frequencies, which are then transferred to the GPU memory. Comparison of vectors against pre-built Markov models is done in the GPUs. The minimum scores are then output to the CPU, and the input sequence will be annotated with the NCBI taxonomy information in the CPU.

MetaBinG is implemented in C with CUBLAS library. The system design of MetaBinG is shown in Figure 2. It has been tested in a Linux machine with 2 Intel Xeon E5520 CPUs (8 CPU cores), 16 GB memory and one NVDIA Tesla C1060 GPU card (240 cores). NVIDIA CUDA compiler driver nvcc release 3.0, V0.2.1221 with options “-L/usr/local/cuda/lib64 -lcudart –lcublas” was used to compile the GPU version source code.

## Supporting Information

Table S1 **The Complete list of training and test genomes.** We downloaded 1212 fully sequenced bacterial genomes from the NCBI FTP site (ftp://ftp.ncbi.nih.gov/genomes/Bacteria/) on 14 Dec 2010. Using the NCBI taxonomy, 390 genomes were removed to guarantee that every genus has at least two genomes. The remaining 822 genomes were assigned to training and test groups. Genomes from a species were assigned to one and only one set, either the training set or the test set. In the end, we generated 468 training genomes and 354 test genomes.
(DOC)

Table S2 **Accuracy of Phymm at different ranks.** The accuracy of Phymm was reported at different ranks. The data sets, the software and parameters are all the same as in Table 2.
(DOC)

Table S3 **Accuracy of MetaBinG at different ranks.** The accuracy of MetaBinG was reported at different ranks. The data sets, the software and parameters are all the same as in Table 2.
(DOC)
