# Supplementary material for: MetaBinG: Using GPUs to Accelerate Metagenomic Sequence Classification
Source: PLoS One. 2011 Nov 23;6(11):e25353. doi: 10.1371/journal.pone.0025353 (PMC3223155; doi:10.1371/journal.pone.0025353)
Supplement: Table S1 — The Complete list of training and test genomes. We downloaded 1212 fully sequenced bacterial genomes from the NCBI FTP site (ftp://ftp.ncbi.nih.gov/genomes/Bacteria/) on 14 Dec 2010. Using the NCBI taxonomy, 390 genomes were removed to guarantee that every genus has at least two genomes. The remaining 822 genomes were assigned to training and test groups. Genomes from a species were assigned to one and only one set, either the training set or the test set. In the end, we generated 468 training genomes and 354 test genomes. (DOC) [file pone.0025353.s001.doc]

**Table S1. The Complete list of training and test genomes.**

| Class | # | Genome |
| --- | --- | --- |
| Training | 1 | Acidovorax_citrulli_AAC00_1_uid58429 |
|  | 2 | Acidovorax_JS42_uid58427 |
|  | 3 | Acinetobacter_baumannii_AB0057_uid59083 |
|  | 4 | Acinetobacter_baumannii_AB307_0294_uid59271 |
|  | 5 | Acinetobacter_baumannii_ACICU_uid58765 |
|  | 6 | Acinetobacter_baumannii_ATCC_17978_uid58731 |
|  | 7 | Acinetobacter_baumannii_AYE |
|  | 8 | Acinetobacter_baumannii_SDF |
|  | 9 | Acinetobacter_sp_ADP1 |
|  | 10 | Actinobacillus_pleuropneumoniae_serovar_3_JL03_uid58891 |
|  | 11 | Actinobacillus_pleuropneumoniae_serovar_5b_L20_uid58789 |
|  | 12 | Actinobacillus_pleuropneumoniae_serovar_7_AP76_uid59231 |
|  | 13 | Aeromonas_salmonicida_A449_uid58631 |
|  | 14 | Aggregatibacter_actinomycetemcomitans_D11S_1 |
|  | 15 | Agrobacterium_radiobacter_K84_uid58269 |
|  | 16 | Agrobacterium_tumefaciens_C58_uid57865 |
|  | 17 | Alkaliphilus_metalliredigens_QYMF_uid58171 |
|  | 18 | Anabaena_variabilis_ATCC_29413_uid58043 |
|  | 19 | Anaeromyxobacter_dehalogenans_2CP_1_uid58989 |
|  | 20 | Anaeromyxobacter_dehalogenans_2CP_C_uid58135 |
|  | 21 | Anaeromyxobacter_K_uid58953 |
|  | 22 | Anaplasma_marginale_Florida_uid58577 |
|  | 23 | Anaplasma_marginale_Maries_uid57629 |
|  | 24 | Anaplasma_phagocytophilum_HZ_uid57951 |
|  | 25 | Archaeoglobus_profundus_DSM_5631_uid43493 |
|  | 26 | Arcobacter_butzleri_RM4018_uid58557 |
|  | 27 | Arthrobacter_chlorophenolicus_A6_uid58969 |
|  | 28 | Arthrobacter_FB24_uid58141 |
|  | 29 | Bacillus_anthracis__Ames_Ancestor__uid58083 |
|  | 30 | Bacillus_anthracis_A0248_uid59385 |
|  | 31 | Bacillus_anthracis_Ames_uid57909 |
|  | 32 | Bacillus_anthracis_CDC_684_uid59303 |
|  | 33 | Bacillus_anthracis_CI_uid50615 |
|  | 34 | Bacillus_anthracis_Sterne_uid58091 |
|  | 35 | Bacillus_atrophaeus_1942_uid59887 |
|  | 36 | Bacillus_cereus_03BB102_uid59299 |
|  | 37 | Bacillus_cereus_AH187_uid58753 |
|  | 38 | Bacillus_cereus_AH820_uid58751 |
|  | 39 | Bacillus_cereus_ATCC_10987_uid57673 |
|  | 40 | Bacillus_cereus_ATCC_14579_uid57975 |
|  | 41 | Bacillus_cereus_B4264_uid58757 |
|  | 42 | Bacillus_cereus_E33L_uid58103 |
|  | 43 | Bacillus_cereus_G9842_uid58759 |
|  | 44 | Bacillus_cereus_Q1_uid58529 |
|  | 45 | Bacillus_halodurans_C_125_uid57791 |
|  | 46 | Bacillus_pumilus_SAFR_032_uid59017 |
|  | 47 | Bacillus_selenitireducens_MLS10_uid49513 |
|  | 48 | Bacillus_subtilis_168_uid57675 |
|  | 49 | Bacillus_subtilis_spizizenii_W23_uid51879 |
|  | 50 | Bacillus_thuringiensis_Al_Hakam_uid58795 |
|  | 51 | Bacillus_thuringiensis_BMB171_uid49135 |
|  | 52 | Bacillus_thuringiensis_serovar_konkukian_97_27_uid58089 |
|  | 53 | Bacillus_tusciae_DSM_2912_uid48361 |
|  | 54 | Bacteroides_fragilis_NCTC_9343_uid57639 |
|  | 55 | Bacteroides_fragilis_YCH46_uid58195 |
|  | 56 | Bacteroides_vulgatus_ATCC_8482_uid58253 |
|  | 57 | Bartonella_bacilliformis_KC583_uid58533 |
|  | 58 | Bartonella_henselae_Houston_1_uid57745 |
|  | 59 | Bartonella_quintana_Toulouse_uid57635 |
|  | 60 | Bifidobacterium_adolescentis_ATCC_15703_uid58559 |
|  | 61 | Bifidobacterium_bifidum_PRL2010_uid59883 |
|  | 62 | Bifidobacterium_bifidum_S17_uid59545 |
|  | 63 | Bifidobacterium_longum_BBMN68_uid60163 |
|  | 64 | Bifidobacterium_longum_DJO10A_uid58833 |
|  | 65 | Bifidobacterium_longum_infantis_ATCC_15697_uid58677 |
|  | 66 | Bifidobacterium_longum_JDM301_uid49131 |
|  | 67 | Bifidobacterium_longum_NCC2705_uid57939 |
|  | 68 | Blattabacterium__Periplaneta_americana__BPLAN |
|  | 69 | Bordetella_parapertussis_12822_uid57615 |
|  | 70 | Bordetella_pertussis_Tohama_I_uid57617 |
|  | 71 | Bordetella_petrii |
|  | 72 | Borrelia_afzelii_PKo_uid58653 |
|  | 73 | Borrelia_burgdorferi_B31_uid57581 |
|  | 74 | Borrelia_burgdorferi_ZS7_uid59429 |
|  | 75 | Borrelia_garinii_PBi_uid58125 |
|  | 76 | Borrelia_hermsii_DAH_uid59225 |
|  | 77 | Brachyspira_murdochii_DSM_12563_uid48819 |
|  | 78 | Brachyspira_pilosicoli_95_1000_uid50609 |
|  | 79 | Bradyrhizobium_japonicum_USDA_110_uid57599 |
|  | 80 | Bradyrhizobium_ORS278_uid58941 |
|  | 81 | Brucella_melitensis_ATCC_23457_uid59241 |
|  | 82 | Brucella_melitensis_bv__1_16M_uid57735 |
|  | 83 | Brucella_microti_CCM_4915_uid59319 |
|  | 84 | Brucella_suis_1330_uid57927 |
|  | 85 | Brucella_suis_ATCC_23445_uid59015 |
|  | 86 | Burkholderia_383_uid58073 |
|  | 87 | Burkholderia_CCGE1002_uid42523 |
|  | 88 | Burkholderia_glumae_BGR1_uid59397 |
|  | 89 | Burkholderia_multivorans_ATCC_17616_uid58697 |
|  | 90 | Burkholderia_multivorans_ATCC_17616_uid58909 |
|  | 91 | Burkholderia_pseudomallei_1106a_uid58515 |
|  | 92 | Burkholderia_pseudomallei_1710b_uid58391 |
|  | 93 | Burkholderia_pseudomallei_668_uid58389 |
|  | 94 | Burkholderia_pseudomallei_K96243_uid57733 |
|  | 95 | Burkholderia_pseudomallei_MSHR346 |
|  | 96 | Burkholderia_thailandensis_E264_uid58081 |
|  | 97 | Burkholderia_vietnamiensis_G4_uid58075 |
|  | 98 | Caldicellulosiruptor_bescii_DSM_6725_uid59201 |
|  | 99 | Caldicellulosiruptor_obsidiansis_OB47_uid51501 |
|  | 100 | Caldicellulosiruptor_owensensis_OL_uid60165 |
|  | 101 | Campylobacter_fetus_82_40_uid58545 |
|  | 102 | Campylobacter_hominis_ATCC_BAA_381_uid58981 |
|  | 103 | Campylobacter_jejuni_81_176_uid58503 |
|  | 104 | Campylobacter_jejuni_81116_uid58771 |
|  | 105 | Campylobacter_jejuni_doylei_269_97_uid58671 |
|  | 106 | Campylobacter_jejuni_NCTC_11168_uid57587 |
|  | 107 | Campylobacter_jejuni_RM1221_uid57899 |
|  | 108 | Candidatus_Blochmannia_floridanus_uid57999 |
|  | 109 | Candidatus_Phytoplasma_australiense |
|  | 110 | Candidatus_Phytoplasma_mali_uid59087 |
|  | 111 | Caulobacter_crescentus_CB15_uid57891 |
|  | 112 | Caulobacter_crescentus_NA1000_uid59307 |
|  | 113 | Caulobacter_K31_uid58551 |
|  | 114 | Chlamydia_trachomatis_434_Bu |
|  | 115 | Chlamydia_trachomatis_A_HAR_13_uid58333 |
|  | 116 | Chlamydia_trachomatis_B_Jali20_OT_uid59351 |
|  | 117 | Chlamydia_trachomatis_B_TZ1A828_OT_uid59349 |
|  | 118 | Chlamydia_trachomatis_D_UW_3_CX_uid57637 |
|  | 119 | Chlamydia_trachomatis_L2b_UCH_1_proctitis |
|  | 120 | Chlamydophila_abortus_S26_3_uid57963 |
|  | 121 | Chlamydophila_caviae_GPIC_uid57783 |
|  | 122 | Chlorobium_chlorochromatii_CaD3_uid58375 |
|  | 123 | Chlorobium_limicola_DSM_245_uid58127 |
|  | 124 | Chlorobium_tepidum_TLS_uid57897 |
|  | 125 | Chloroflexus_aurantiacus_J_10_fl_uid57657 |
|  | 126 | Chloroflexus_Y_400_fl_uid59085 |
|  | 127 | Citrobacter_koseri_ATCC_BAA_895_uid58143 |
|  | 128 | Clostridiales_genomosp__BVAB3_UPII9_5_uid46219 |
|  | 129 | Clostridium_beijerinckii_NCIMB_8052_uid58137 |
|  | 130 | Clostridium_botulinum_A |
|  | 131 | Clostridium_botulinum_A_ATCC_19397_uid58927 |
|  | 132 | Clostridium_botulinum_A_Hall_uid58931 |
|  | 133 | Clostridium_botulinum_A2_Kyoto_uid59229 |
|  | 134 | Clostridium_botulinum_A3_Loch_Maree_uid59149 |
|  | 135 | Clostridium_botulinum_B_Eklund_17B_uid59159 |
|  | 136 | Clostridium_botulinum_B1_Okra_uid59147 |
|  | 137 | Clostridium_botulinum_Ba4_657_uid59173 |
|  | 138 | Clostridium_botulinum_E3_Alaska_E43_uid59157 |
|  | 139 | Clostridium_botulinum_F_Langeland_uid58929 |
|  | 140 | Clostridium_cellulolyticum_H10_uid58709 |
|  | 141 | Clostridium_cellulovorans_743B_uid51503 |
|  | 142 | Clostridium_kluyveri_DSM_555_uid58885 |
|  | 143 | Clostridium_kluyveri_NBRC_12016_uid59369 |
|  | 144 | Clostridium_phytofermentans_ISDg_uid58519 |
|  | 145 | Clostridium_saccharolyticum_WM1_uid51419 |
|  | 146 | Clostridium_sticklandii_DSM_519_uid59585 |
|  | 147 | Corynebacterium_diphtheriae_NCTC_13129_uid57691 |
|  | 148 | Corynebacterium_glutamicum_ATCC_13032_Bielefeld |
|  | 149 | Corynebacterium_glutamicum_ATCC_13032_uid57905 |
|  | 150 | Corynebacterium_glutamicum_R_uid58897 |
|  | 151 | Corynebacterium_jeikeium_K411_uid58399 |
|  | 152 | Corynebacterium_urealyticum_DSM_7109 |
|  | 153 | Cronobacter_sakazakii_ATCC_BAA_894_uid58145 |
|  | 154 | Cupriavidus_metallidurans_CH34_uid57815 |
|  | 155 | Cyanothece_ATCC_51142_uid59013 |
|  | 156 | Cyanothece_PCC_7425_uid59435 |
|  | 157 | Cyanothece_PCC_8801_uid59027 |
|  | 158 | Dehalococcoides_BAV1_uid58477 |
|  | 159 | Dehalococcoides_CBDB1_uid58413 |
|  | 160 | Dehalococcoides_GT_uid42115 |
|  | 161 | Deinococcus_deserti_VCD115_uid58615 |
|  | 162 | Deinococcus_radiodurans_R1_uid57665 |
|  | 163 | Desulfotomaculum_acetoxidans_DSM_771_uid59109 |
|  | 164 | Desulfovibrio_desulfuricans_ATCC_27774_uid59213 |
|  | 165 | Desulfovibrio_desulfuricans_G20_uid57941 |
|  | 166 | Desulfovibrio_vulgaris__Miyazaki_F__uid59089 |
|  | 167 | Desulfovibrio_vulgaris_DP4_uid58679 |
|  | 168 | Desulfovibrio_vulgaris_Hildenborough_uid57645 |
|  | 169 | Dickeya_dadantii_3937_uid52537 |
|  | 170 | Dickeya_dadantii_Ech586 |
|  | 171 | Dickeya_dadantii_Ech703_uid59363 |
|  | 172 | Dictyoglomus_turgidum_DSM_6724_uid59177 |
|  | 173 | Edwardsiella_ictaluri_93_146_uid59403 |
|  | 174 | Ehrlichia_canis_Jake_uid58071 |
|  | 175 | Ehrlichia_ruminantium_Gardel_uid58245 |
|  | 176 | Ehrlichia_ruminantium_Welgevonden_uid58013 |
|  | 177 | Ehrlichia_ruminantium_Welgevonden_uid58243 |
|  | 178 | Enterobacter_cloacae_ATCC_13047_uid48363 |
|  | 179 | Enterobacter_cloacae_SCF1_uid59969 |
|  | 180 | Erwinia_amylovora_ATCC_49946_uid46943 |
|  | 181 | Erwinia_amylovora_CFBP1430_uid46839 |
|  | 182 | Erwinia_billingiae_Eb661_uid50547 |
|  | 183 | Escherichia_coli__BL21_Gold_DE3_pLysS_AG__uid59245 |
|  | 184 | Escherichia_coli_536_uid58531 |
|  | 185 | Escherichia_coli_55989_uid59383 |
|  | 186 | Escherichia_coli_APEC_O1_uid58623 |
|  | 187 | Escherichia_coli_ATCC_8739_uid58783 |
|  | 188 | Escherichia_coli_B_REL606_uid58803 |
|  | 189 | Escherichia_coli_BW2952_uid59391 |
|  | 190 | Escherichia_coli_CFT073_uid57915 |
|  | 191 | Escherichia_coli_E24377A_uid58395 |
|  | 192 | Escherichia_coli_ED1a_uid59379 |
|  | 193 | Escherichia_coli_HS_uid58393 |
|  | 194 | Escherichia_coli_IAI1_uid59377 |
|  | 195 | Escherichia_coli_IAI39_uid59381 |
|  | 196 | Escherichia_coli_K_12_substr__DH10B_uid58979 |
|  | 197 | Escherichia_coli_K_12_substr__MG1655_uid57779 |
|  | 198 | Escherichia_coli_K_12_substr__W3110_uid58567 |
|  | 199 | Escherichia_coli_O103_H2_12009 |
|  | 200 | Escherichia_coli_O111_H__11128 |
|  | 201 | Escherichia_coli_O127_H6_E2348_69_uid59343 |
|  | 202 | Escherichia_coli_O157_H7_EC4115_uid59091 |
|  | 203 | Escherichia_coli_O157_H7_EDL933_uid57831 |
|  | 204 | Escherichia_coli_O157_H7_Sakai_uid57781 |
|  | 205 | Escherichia_coli_O157_H7_TW14359_uid59235 |
|  | 206 | Escherichia_coli_O26_H11_11368 |
|  | 207 | Escherichia_coli_O55_H7_CB9615_uid46655 |
|  | 208 | Escherichia_coli_S88 |
|  | 209 | Escherichia_coli_SE11_uid59425 |
|  | 210 | Escherichia_coli_SMS_3_5_uid58919 |
|  | 211 | Escherichia_coli_UMN026 |
|  | 212 | Escherichia_coli_UTI89_uid58541 |
|  | 213 | Eubacterium_eligens_ATCC_27750_uid59171 |
|  | 214 | Eubacterium_limosum_KIST612_uid59777 |
|  | 215 | Exiguobacterium_AT1b_uid59093 |
|  | 216 | Flavobacteriaceae_bacterium_3519_10_uid59413 |
|  | 217 | Flavobacteriales_bacterium_HTCC2170_uid51877 |
|  | 218 | Flavobacterium_psychrophilum_JIP02_86 |
|  | 219 | Francisella_novicida_U112_uid58499 |
|  | 220 | Francisella_philomiragia_ATCC_25017_uid59105 |
|  | 221 | Frankia_alni_ACN14a_uid58695 |
|  | 222 | Frankia_CcI3_uid58397 |
|  | 223 | gamma_proteobacterium_HdN1_uid51635 |
|  | 224 | Geobacillus_C56_T3_uid49467 |
|  | 225 | Geobacillus_kaustophilus_HTA426_uid58227 |
|  | 226 | Geobacillus_Y412MC10 |
|  | 227 | Geobacillus_Y412MC61 |
|  | 228 | Geobacter_bemidjiensis_Bem_uid58749 |
|  | 229 | Geobacter_metallireducens_GS_15_uid57731 |
|  | 230 | Geobacter_sulfurreducens_PCA_uid57743 |
|  | 231 | Geobacter_uraniireducens_Rf4_uid58475 |
|  | 232 | Haemophilus_ducreyi_35000HP_uid57625 |
|  | 233 | Haemophilus_influenzae_86_028NP_uid58093 |
|  | 234 | Haemophilus_influenzae_PittEE_uid58591 |
|  | 235 | Haemophilus_influenzae_PittGG_uid58593 |
|  | 236 | Haemophilus_influenzae_Rd_KW20_uid57771 |
|  | 237 | Helicobacter_hepaticus_ATCC_51449_uid57737 |
|  | 238 | Helicobacter_pylori_26695_uid57787 |
|  | 239 | Helicobacter_pylori_B38_uid59415 |
|  | 240 | Helicobacter_pylori_B8_uid49873 |
|  | 241 | Helicobacter_pylori_G27_uid59305 |
|  | 242 | Helicobacter_pylori_HPAG1_uid58517 |
|  | 243 | Helicobacter_pylori_J99_uid57789 |
|  | 244 | Helicobacter_pylori_P12_uid59327 |
|  | 245 | Helicobacter_pylori_PeCan4_uid53539 |
|  | 246 | Helicobacter_pylori_Shi470_uid59165 |
|  | 247 | Helicobacter_pylori_SJM180_uid53541 |
|  | 248 | Klebsiella_pneumoniae_342_uid59145 |
|  | 249 | Klebsiella_pneumoniae_MGH_78578_uid57619 |
|  | 250 | Klebsiella_pneumoniae_NTUH_K2044_uid59073 |
|  | 251 | Lactobacillus_acidophilus_NCFM_uid57685 |
|  | 252 | Lactobacillus_brevis_ATCC_367_uid57989 |
|  | 253 | Lactobacillus_casei_ATCC_334_uid57985 |
|  | 254 | Lactobacillus_casei_BL23_uid59237 |
|  | 255 | Lactobacillus_casei_Zhang_uid50673 |
|  | 256 | Lactobacillus_fermentum_IFO_3956_uid58865 |
|  | 257 | Lactobacillus_johnsonii_FI9785 |
|  | 258 | Lactobacillus_johnsonii_NCC_533_uid58029 |
|  | 259 | Lactobacillus_plantarum |
|  | 260 | Lactobacillus_plantarum_JDM1_uid59361 |
|  | 261 | Lactobacillus_plantarum_ST_III_uid53537 |
|  | 262 | Lactobacillus_rhamnosus_GG_uid59313 |
|  | 263 | Lactobacillus_rhamnosus_Lc_705_uid59315 |
|  | 264 | Legionella_pneumophila_2300_99_Alcoy_uid48801 |
|  | 265 | Legionella_pneumophila_Corby_uid58733 |
|  | 266 | Legionella_pneumophila_Lens_uid58209 |
|  | 267 | Legionella_pneumophila_Paris_uid58211 |
|  | 268 | Legionella_pneumophila_Philadelphia_1_uid57609 |
|  | 269 | Leptospira_borgpetersenii_serovar_Hardjo_bovis_JB197_uid58509 |
|  | 270 | Leptospira_borgpetersenii_serovar_Hardjo_bovis_L550_uid58507 |
|  | 271 | Leptospira_interrogans_serovar_Copenhageni_Fiocruz_L1_130_uid58065 |
|  | 272 | Leptospira_interrogans_serovar_Lai_56601_uid57881 |
|  | 273 | Leuconostoc_citreum_KM20_uid58481 |
|  | 274 | Leuconostoc_mesenteroides_ATCC_8293_uid57919 |
|  | 275 | Listeria_monocytogenes |
|  | 276 | Listeria_monocytogenes_08_5923_uid43727 |
|  | 277 | Listeria_monocytogenes_Clip80459_uid59317 |
|  | 278 | Listeria_monocytogenes_HCC23_uid59203 |
|  | 279 | Listeria_monocytogenes_serotype_4b_F2365_uid57689 |
|  | 280 | Listeria_monocytogenes_uid43671 |
|  | 281 | Listeria_seeligeri_serovar_1_2b_SLCC3954_uid46215 |
|  | 282 | Meiothermus_ruber_DSM_1279_uid46661 |
|  | 283 | Methanobrevibacter_smithii_ATCC_35061_uid58827 |
|  | 284 | Methanocaldococcus_fervens_AG86_uid59347 |
|  | 285 | Methanocaldococcus_jannaschii_DSM_2661_uid57713 |
|  | 286 | Methanocaldococcus_vulcanius_M7 |
|  | 287 | Methanococcus_aeolicus_Nankai_3_uid58823 |
|  | 288 | Methanococcus_maripaludis_C5_uid58741 |
|  | 289 | Methanococcus_maripaludis_C6_uid58947 |
|  | 290 | Methanococcus_maripaludis_C7_uid58847 |
|  | 291 | Methanococcus_maripaludis_S2_uid58035 |
|  | 292 | Methanosarcina_barkeri_Fusaro_uid57715 |
|  | 293 | Methanosarcina_mazei_Go1_uid57893 |
|  | 294 | Methanothermobacter_thermautotrophicus_Delta_H_uid57877 |
|  | 295 | Methylobacterium_4_46_uid58843 |
|  | 296 | Methylobacterium_extorquens_AM1_uid57605 |
|  | 297 | Methylobacterium_extorquens_DM4 |
|  | 298 | Methylobacterium_extorquens_PA1_uid58821 |
|  | 299 | Methylobacterium_nodulans_ORS_2060_uid59023 |
|  | 300 | Methylotenera_mobilis_JLW8_uid59373 |
|  | 301 | Mycobacterium_abscessus_ATCC_19977 |
|  | 302 | Mycobacterium_avium_104_uid57693 |
|  | 303 | Mycobacterium_avium_paratuberculosis_K_10_uid57699 |
|  | 304 | Mycobacterium_JLS_uid58489 |
|  | 305 | Mycobacterium_leprae_Br4923_uid59293 |
|  | 306 | Mycobacterium_leprae_TN_uid57697 |
|  | 307 | Mycobacterium_smegmatis_MC2_155_uid57701 |
|  | 308 | Mycobacterium_ulcerans_Agy99 |
|  | 309 | Mycobacterium_vanbaalenii_PYR_1_uid58463 |
|  | 310 | Mycoplasma_arthritidis_158L3_1_uid58005 |
|  | 311 | Mycoplasma_conjunctivae_HRC_581_uid59325 |
|  | 312 | Mycoplasma_crocodyli_MP145_uid47087 |
|  | 313 | Mycoplasma_fermentans_JER_uid53543 |
|  | 314 | Mycoplasma_genitalium_G37_uid57707 |
|  | 315 | Mycoplasma_hominis |
|  | 316 | Mycoplasma_hyopneumoniae_232_uid58205 |
|  | 317 | Mycoplasma_hyopneumoniae_7448_uid58039 |
|  | 318 | Mycoplasma_hyopneumoniae_J_uid58059 |
|  | 319 | Mycoplasma_mycoides_SC_PG1_uid58031 |
|  | 320 | Mycoplasma_pulmonis |
|  | 321 | Neisseria_gonorrhoeae_FA_1090_uid57611 |
|  | 322 | Neisseria_gonorrhoeae_NCCP11945_uid59191 |
|  | 323 | Neorickettsia_risticii_Illinois_uid58889 |
|  | 324 | Nitrobacter_hamburgensis_X14_uid58293 |
|  | 325 | Nitrosococcus_oceani_ATCC_19707_uid58403 |
|  | 326 | Nitrosococcus_watsoni_C_113_uid50331 |
|  | 327 | Nitrosomonas_eutropha_C91_uid58363 |
|  | 328 | Nostoc_punctiforme_PCC_73102_uid57767 |
|  | 329 | Paenibacillus_JDR_2_uid59021 |
|  | 330 | Pantoea_vagans_C9_1_uid49871 |
|  | 331 | Pectobacterium_carotovorum_PC1_uid59295 |
|  | 332 | Pectobacterium_wasabiae_WPP163 |
|  | 333 | Pelobacter_carbinolicus_DSM_2380_uid58241 |
|  | 334 | Pelodictyon_phaeoclathratiforme_BU_1_uid58173 |
|  | 335 | Photorhabdus_luminescens |
|  | 336 | Polaromonas_JS666_uid58207 |
|  | 337 | Prevotella_melaninogenica_ATCC_25845_uid51377 |
|  | 338 | Propionibacterium_freudenreichii_shermanii_CIRM_BIA1_uid49535 |
|  | 339 | Pseudoalteromonas_haloplanktis_TAC125_uid58431 |
|  | 340 | Pseudomonas_aeruginosa_LESB58_uid59275 |
|  | 341 | Pseudomonas_aeruginosa_PA7_uid58627 |
|  | 342 | Pseudomonas_aeruginosa_PAO1_uid57945 |
|  | 343 | Pseudomonas_aeruginosa_UCBPP_PA14_uid57977 |
|  | 344 | Pseudomonas_mendocina_ymp_uid58723 |
|  | 345 | Pseudomonas_stutzeri_A1501_uid58641 |
|  | 346 | Pseudomonas_syringae_phaseolicola_1448A_uid58099 |
|  | 347 | Pseudomonas_syringae_tomato_DC3000_uid57967 |
|  | 348 | Psychrobacter_arcticus_273_4_uid58021 |
|  | 349 | Psychrobacter_PRwf_1_uid58459 |
|  | 350 | Pyrobaculum_aerophilum_IM2_uid57727 |
|  | 351 | Pyrobaculum_arsenaticum_DSM_13514_uid58409 |
|  | 352 | Pyrococcus_abyssi |
|  | 353 | Pyrococcus_horikoshii_OT3_uid57753 |
|  | 354 | Ralstonia_eutropha_JMP134_uid58047 |
|  | 355 | Ralstonia_solanacearum_CFBP2957_uid50545 |
|  | 356 | Ralstonia_solanacearum_GMI1000_uid57593 |
|  | 357 | Ralstonia_solanacearum_PSI07_uid50539 |
|  | 358 | Rhizobium_etli_CFN_42_uid58377 |
|  | 359 | Rhizobium_etli_CIAT_652_uid59115 |
|  | 360 | Rhizobium_NGR234_uid59081 |
|  | 361 | Rhodobacter_sphaeroides_2_4_1_uid57653 |
|  | 362 | Rhodobacter_sphaeroides_ATCC_17025_uid58451 |
|  | 363 | Rhodobacter_sphaeroides_ATCC_17029_uid58449 |
|  | 364 | Rhodobacter_sphaeroides_KD131_uid59277 |
|  | 365 | Rhodococcus_equi_103S_uid60171 |
|  | 366 | Rhodococcus_opacus_B4_uid13791 |
|  | 367 | Rhodospirillum_centenum_SW_uid58805 |
|  | 368 | Rickettsia_akari_Hartford_uid58161 |
|  | 369 | Rickettsia_bellii_OSU_85_389_uid58681 |
|  | 370 | Rickettsia_bellii_RML369_C_uid58405 |
|  | 371 | Rickettsia_felis_URRWXCal2_uid58331 |
|  | 372 | Rickettsia_massiliae_MTU5_uid58801 |
|  | 373 | Rickettsia_rickettsii__Sheila_Smith__uid58027 |
|  | 374 | Rickettsia_rickettsii_Iowa_uid58961 |
|  | 375 | Rickettsia_typhi_Wilmington_uid58063 |
|  | 376 | Roseiflexus_castenholzii_DSM_13941_uid58287 |
|  | 377 | Rothia_mucilaginosa |
|  | 378 | Ruegeria_pomeroyi_DSS_3_uid57863 |
|  | 379 | Salinispora_tropica_CNB_440_uid58565 |
|  | 380 | Shewanella_amazonensis_SB2B_uid58257 |
|  | 381 | Shewanella_baltica_OS155_uid58259 |
|  | 382 | Shewanella_baltica_OS185_uid58743 |
|  | 383 | Shewanella_baltica_OS195_uid58261 |
|  | 384 | Shewanella_baltica_OS223_uid58775 |
|  | 385 | Shewanella_halifaxensis_HAW_EB4_uid59007 |
|  | 386 | Shewanella_loihica_PV_4_uid58349 |
|  | 387 | Shewanella_MR_4_uid58345 |
|  | 388 | Shewanella_MR_7_uid58343 |
|  | 389 | Shewanella_oneidensis_MR_1_uid57949 |
|  | 390 | Shewanella_violacea_DSS12_uid47085 |
|  | 391 | Shewanella_W3_18_1_uid58341 |
|  | 392 | Shigella_boydii_CDC_3083_94_uid58415 |
|  | 393 | Shigella_boydii_Sb227_uid58215 |
|  | 394 | Shigella_flexneri_2a |
|  | 395 | Shigella_flexneri_2a_2457T_uid57991 |
|  | 396 | Shigella_flexneri_5_8401_uid58583 |
|  | 397 | Sinorhizobium_meliloti_1021_uid57603 |
|  | 398 | Spirochaeta_smaragdinae_DSM_11293_uid51369 |
|  | 399 | Staphylococcus_carnosus_TM300_uid59401 |
|  | 400 | Staphylococcus_haemolyticus |
|  | 401 | Staphylococcus_saprophyticus_ATCC_15305_uid58411 |
|  | 402 | Staphylothermus_hellenicus_DSM_12710_uid45893 |
|  | 403 | Streptococcus_agalactiae_2603V_R_uid57943 |
|  | 404 | Streptococcus_agalactiae_A909_uid57935 |
|  | 405 | Streptococcus_agalactiae_NEM316 |
|  | 406 | Streptococcus_dysgalactiae_equisimilis_GGS_124_uid59103 |
|  | 407 | Streptococcus_equi_4047_uid59259 |
|  | 408 | Streptococcus_equi_zooepidemicus_MGCS10565_uid59263 |
|  | 409 | Streptococcus_equi_zooepidemicus_uid59261 |
|  | 410 | Streptococcus_gallolyticus_UCN34_uid46061 |
|  | 411 | Streptococcus_gordonii_Challis_substr__CH1_uid57667 |
|  | 412 | Streptococcus_mitis_B6_uid46097 |
|  | 413 | Streptococcus_uberis_0140J_uid57959 |
|  | 414 | Streptomyces_coelicolor_A3_2__uid57801 |
|  | 415 | Streptomyces_griseus_NBRC_13350_uid58983 |
|  | 416 | Sulfolobus_islandicus_L_D_8_5_uid43679 |
|  | 417 | Sulfolobus_islandicus_L_S_2_15_uid58871 |
|  | 418 | Sulfolobus_islandicus_M_14_25_uid58849 |
|  | 419 | Sulfolobus_islandicus_M_16_27_uid58851 |
|  | 420 | Sulfolobus_islandicus_M_16_4_uid58841 |
|  | 421 | Sulfolobus_islandicus_Y_G_57_14_uid58923 |
|  | 422 | Sulfolobus_islandicus_Y_N_15_51_uid58825 |
|  | 423 | Sulfolobus_solfataricus_P2_uid57721 |
|  | 424 | Sulfurihydrogenibium_YO3AOP1_uid58855 |
|  | 425 | Sulfurimonas_autotrophica_DSM_16294_uid53043 |
|  | 426 | Synechococcus_CC9311_uid58123 |
|  | 427 | Synechococcus_CC9605_uid58319 |
|  | 428 | Synechococcus_RCC307 |
|  | 429 | Synechococcus_sp_WH8102 |
|  | 430 | Synechococcus_WH_7803 |
|  | 431 | Thermoanaerobacter_mathranii_A3_uid49481 |
|  | 432 | Thermoanaerobacter_X513_uid53065 |
|  | 433 | Thermoanaerobacter_X514_uid58589 |
|  | 434 | Thermococcus_onnurineus_NA1_uid59043 |
|  | 435 | Thermococcus_sibiricus_MM_739_uid59399 |
|  | 436 | Thermoplasma_acidophilum |
|  | 437 | Thermosipho_melanesiensis_BI429_uid58683 |
|  | 438 | Thermotoga_neapolitana_DSM_4359_uid59065 |
|  | 439 | Thermotoga_petrophila_RKU_1_uid58655 |
|  | 440 | Thermotoga_RQ2_uid58935 |
|  | 441 | Thioalkalivibrio_K90mix_uid46181 |
|  | 442 | Treponema_pallidum_Nichols_uid57585 |
|  | 443 | Treponema_pallidum_SS14_uid58977 |
|  | 444 | uncultured_methanogenic_archaeon_RC-I |
|  | 445 | Ureaplasma_parvum_serovar_3_ATCC_27815_uid58887 |
|  | 446 | Ureaplasma_parvum_serovar_3_ATCC_700970_uid57711 |
|  | 447 | Vibrio_fischeri_ES114_uid58163 |
|  | 448 | Vibrio_fischeri_MJ11_uid58907 |
|  | 449 | Vibrio_harveyi_ATCC_BAA_1116_uid58957 |
|  | 450 | Vibrio_parahaemolyticus_RIMD_2210633_uid57969 |
|  | 451 | Vibrio_splendidus_LGP32_uid59353 |
|  | 452 | Wolbachia_endosymbiont_of_Culex_quinquefasciatus_Pel |
|  | 453 | Wolbachia_wRi_uid59371 |
|  | 454 | Xanthomonas_axonopodis_citri_306_uid57889 |
|  | 455 | Xanthomonas_campestris_vesicatoria_85_10_uid58321 |
|  | 456 | Xanthomonas_oryzae_KACC10331_uid58155 |
|  | 457 | Xanthomonas_oryzae_MAFF_311018_uid58547 |
|  | 458 | Xanthomonas_oryzae_PXO99A_uid59131 |
|  | 459 | Xenorhabdus_nematophila_ATCC_19061_uid49133 |
|  | 460 | Yersinia_enterocolitica_8081_uid57741 |
|  | 461 | Yersinia_pestis_Angola_uid58485 |
|  | 462 | Yersinia_pestis_Antiqua_uid58607 |
|  | 463 | Yersinia_pestis_biovar_Microtus_91001_uid58037 |
|  | 464 | Yersinia_pestis_CO92_uid57621 |
|  | 465 | Yersinia_pestis_KIM_10_uid57875 |
|  | 466 | Yersinia_pestis_Nepal516_uid58609 |
|  | 467 | Yersinia_pestis_Pestoides_F_uid58619 |
|  | 468 | Yersinia_pestis_Z176003_uid47317 |
|  |  |  |
| Test | 1 | _Nostoc_azollae__0708_uid49725 |
|  | 2 | Acidovorax_ebreus_TPSY_uid59233 |
|  | 3 | Acinetobacter_DR1_uid50119 |
|  | 4 | Actinobacillus_succinogenes_130Z_uid58247 |
|  | 5 | Aeromonas_hydrophila_ATCC_7966_uid58617 |
|  | 6 | Aggregatibacter_aphrophilus_NJ8700_uid59407 |
|  | 7 | Agrobacterium_vitis_S4_uid58249 |
|  | 8 | Aliivibrio_salmonicida_LFI1238_uid59251 |
|  | 9 | Alkaliphilus_oremlandii_OhILAs_uid58495 |
|  | 10 | Anaeromyxobacter_Fw109_5_uid58755 |
|  | 11 | Anaplasma_centrale_Israel |
|  | 12 | Archaeoglobus_fulgidus_DSM_4304_uid57717 |
|  | 13 | Arcobacter_nitrofigilis_DSM_7299_uid49001 |
|  | 14 | Arthrobacter_arilaitensis_Re117_uid53509 |
|  | 15 | Arthrobacter_aurescens_TC1_uid58109 |
|  | 16 | Aster_yellows_witches_broom_phytoplasma_AYWB_uid58297 |
|  | 17 | Bacillus_amyloliquefaciens_DSM7_uid53535 |
|  | 18 | Bacillus_amyloliquefaciens_FZB42_uid58271 |
|  | 19 | Bacillus_clausii_KSM_K16_uid58237 |
|  | 20 | Bacillus_cytotoxicus_NVH_391_98_uid58317 |
|  | 21 | Bacillus_licheniformis_ATCC_14580_uid58097 |
|  | 22 | Bacillus_licheniformis_ATCC_14580_uid58199 |
|  | 23 | Bacillus_megaterium_DSM319_uid48371 |
|  | 24 | Bacillus_megaterium_QM_B1551_uid15862 |
|  | 25 | Bacillus_pseudofirmus_OF4_uid45847 |
|  | 26 | Bacillus_weihenstephanensis_KBAB4_uid58315 |
|  | 27 | Bacteroides_thetaiotaomicron_VPI-5482 |
|  | 28 | Bartonella_grahamii_as4aup_uid59405 |
|  | 29 | Bartonella_tribocorum_CIP_105476_uid59129 |
|  | 30 | Baumannia_cicadellinicola_Hc__Homalodisca_coagulata__uid58111 |
|  | 31 | Bifidobacterium_animalis_lactis_AD011_uid58911 |
|  | 32 | Bifidobacterium_animalis_lactis_Bl_04_uid59359 |
|  | 33 | Bifidobacterium_animalis_lactis_DSM_10140_uid59357 |
|  | 34 | Bifidobacterium_dentium_Bd1 |
|  | 35 | Blattabacterium__Blattella_germanica__Bge |
|  | 36 | Bordetella_avium_197N |
|  | 37 | Bordetella_bronchiseptica_RB50_uid57613 |
|  | 38 | Borrelia_duttonii_Ly_uid58791 |
|  | 39 | Borrelia_recurrentis_A1_uid58793 |
|  | 40 | Borrelia_turicatae_91E135_uid58311 |
|  | 41 | Brachyspira_hyodysenteriae_WA1_uid59291 |
|  | 42 | Bradyrhizobium_BTAi1_uid58505 |
|  | 43 | Brucella_abortus_bv__1_9_941_uid58019 |
|  | 44 | Brucella_abortus_S19_uid58873 |
|  | 45 | Brucella_canis_ATCC_23365_uid59009 |
|  | 46 | Brucella_melitensis_biovar_Abortus |
|  | 47 | Brucella_ovis_ATCC_25840_uid58113 |
|  | 48 | Burkholderia_ambifaria_AMMD_uid58303 |
|  | 49 | Burkholderia_ambifaria_MC40_6_uid58701 |
|  | 50 | Burkholderia_CCGE1003_uid46253 |
|  | 51 | Burkholderia_cenocepacia_AU_1054_uid58371 |
|  | 52 | Burkholderia_cenocepacia_HI2424_uid58369 |
|  | 53 | Burkholderia_cenocepacia_J2315_uid57953 |
|  | 54 | Burkholderia_cenocepacia_MC0_3_uid58769 |
|  | 55 | Burkholderia_mallei_ATCC_23344_uid57725 |
|  | 56 | Burkholderia_mallei_NCTC_10229_uid58383 |
|  | 57 | Burkholderia_mallei_NCTC_10247_uid58385 |
|  | 58 | Burkholderia_mallei_SAVP1_uid58387 |
|  | 59 | Burkholderia_phymatum_STM815_uid58699 |
|  | 60 | Burkholderia_phytofirmans_PsJN_uid58729 |
|  | 61 | Burkholderia_xenovorans_LB400_uid57823 |
|  | 62 | Caldicellulosiruptor_hydrothermalis_108_uid60157 |
|  | 63 | Caldicellulosiruptor_saccharolyticus_DSM_8903_uid58289 |
|  | 64 | Campylobacter_concisus_13826_uid58667 |
|  | 65 | Campylobacter_curvus_525_92_uid58669 |
|  | 66 | Campylobacter_lari_RM2100_uid58115 |
|  | 67 | Candidatus_Blochmannia_pennsylvanicus_BPEN_uid58329 |
|  | 68 | Candidatus_Ruthia_magnifica_Cm__Calyptogena_magnifica__uid58645 |
|  | 69 | Candidatus_Vesicomyosocius_okutanii_HA_uid59427 |
|  | 70 | Caulobacter_segnis_ATCC_21756_uid41709 |
|  | 71 | Chlamydia_muridarum_Nigg_uid57785 |
|  | 72 | Chlamydophila_felis_Fe_C_56_uid57971 |
|  | 73 | Chlamydophila_pneumoniae_CWL029_uid57811 |
|  | 74 | Chlamydophila_pneumoniae_J138_uid57829 |
|  | 75 | Chlamydophila_pneumoniae_TW_183_uid57997 |
|  | 76 | Chlorobaculum_parvum_NCIB_8327_uid59185 |
|  | 77 | Chlorobium_luteolum_DSM_273_uid58175 |
|  | 78 | Chlorobium_phaeobacteroides_BS1_uid58131 |
|  | 79 | Chlorobium_phaeobacteroides_DSM_266_uid58133 |
|  | 80 | Chlorobium_phaeovibrioides_DSM_265_uid58129 |
|  | 81 | Chloroflexus_aggregans_DSM_9485_uid58621 |
|  | 82 | Citrobacter_rodentium_ICC168 |
|  | 83 | Clostridium_acetobutylicum_ATCC_824_uid57677 |
|  | 84 | Clostridium_difficile_630_uid57679 |
|  | 85 | Clostridium_difficile_CD196 |
|  | 86 | Clostridium_difficile_R20291 |
|  | 87 | Clostridium_ljungdahlii_DSM_13528_uid50583 |
|  | 88 | Clostridium_novyi_NT_uid58643 |
|  | 89 | Clostridium_perfringens_13_uid57681 |
|  | 90 | Clostridium_perfringens_ATCC_13124_uid57901 |
|  | 91 | Clostridium_perfringens_SM101_uid58117 |
|  | 92 | Clostridium_tetani_E88_uid57683 |
|  | 93 | Clostridium_thermocellum_ATCC_27405_uid57917 |
|  | 94 | Corynebacterium_aurimucosum_ATCC_700975_uid59409 |
|  | 95 | Corynebacterium_efficiens_YS-314 |
|  | 96 | Corynebacterium_kroppenstedtii_DSM_44385_uid59411 |
|  | 97 | Corynebacterium_pseudotuberculosis_uid50585 |
|  | 98 | Cronobacter_turicensis_z3032_uid40821 |
|  | 99 | Cupriavidus_taiwanensis |
|  | 100 | cyanobacterium_UCYN_A_uid43697 |
|  | 101 | Cyanothece_PCC_7424_uid59025 |
|  | 102 | Cyanothece_PCC_7822_uid52547 |
|  | 103 | Cyanothece_PCC_8802_uid59143 |
|  | 104 | Dehalococcoides_ethenogenes_195_uid57763 |
|  | 105 | Dehalococcoides_VS |
|  | 106 | Deinococcus_geothermalis_DSM_11300_uid58275 |
|  | 107 | Desulfotomaculum_reducens_MI_1_uid58277 |
|  | 108 | Desulfovibrio_magneticus_RS_1_uid59309 |
|  | 109 | Desulfovibrio_salexigens_DSM_2638_uid59223 |
|  | 110 | Dickeya_zeae_Ech1591_uid59297 |
|  | 111 | Dictyoglomus_thermophilum_H_6_12_uid59439 |
|  | 112 | Edwardsiella_tarda_EIB202 |
|  | 113 | Ehrlichia_chaffeensis_Arkansas_uid57933 |
|  | 114 | Enterobacter_638_uid58727 |
|  | 115 | Erwinia_pyrifoliae_Ep1_96_uid40659 |
|  | 116 | Erwinia_tasmaniensis_Et1_99_uid59029 |
|  | 117 | Escherichia_fergusonii_ATCC_35469_uid59375 |
|  | 118 | Eubacterium_rectale_ATCC_33656_uid59169 |
|  | 119 | Exiguobacterium_sibiricum_255_15_uid58053 |
|  | 120 | Flavobacterium_johnsoniae_UW101_uid58493 |
|  | 121 | Francisella_tularensis_FSC198_uid58693 |
|  | 122 | Francisella_tularensis_holarctica_FTNF002_00_uid58999 |
|  | 123 | Francisella_tularensis_holarctica_LVS_uid58595 |
|  | 124 | Francisella_tularensis_holarctica_OSU18_uid58687 |
|  | 125 | Francisella_tularensis_mediasiatica_FSC147_uid58939 |
|  | 126 | Francisella_tularensis_SCHU_S4_uid57589 |
|  | 127 | Francisella_tularensis_WY96_3418_uid58811 |
|  | 128 | Frankia_EAN1pec_uid58367 |
|  | 129 | Frankia_EuI1c_uid42615 |
|  | 130 | Geobacillus_thermodenitrificans_NG80_2_uid58829 |
|  | 131 | Geobacillus_WCH70_uid59045 |
|  | 132 | Geobacillus_Y4_1MC1_uid55779 |
|  | 133 | Geobacter_FRC_32_uid58543 |
|  | 134 | Geobacter_lovleyi_SZ_uid58713 |
|  | 135 | Geobacter_M21_uid59037 |
|  | 136 | Haemophilus_parasuis_SH0165_uid59273 |
|  | 137 | Helicobacter_acinonychis_Sheeba_uid58685 |
|  | 138 | Helicobacter_mustelae_12198_uid46647 |
|  | 139 | Klebsiella_variicola_At_22_uid42113 |
|  | 140 | Lactobacillus_crispatus_ST1_uid48359 |
|  | 141 | Lactobacillus_delbrueckii_bulgaricus_ATCC_11842_uid58647 |
|  | 142 | Lactobacillus_delbrueckii_bulgaricus_ATCC_BAA_365_uid57987 |
|  | 143 | Lactobacillus_gasseri_ATCC_33323_uid57687 |
|  | 144 | Lactobacillus_helveticus_DPC_4571_uid58761 |
|  | 145 | Lactobacillus_reuteri_DSM_20016_uid58471 |
|  | 146 | Lactobacillus_reuteri_JCM_1112_uid58875 |
|  | 147 | Lactobacillus_sakei_23K_uid58281 |
|  | 148 | Lactobacillus_salivarius_UCC118_uid58233 |
|  | 149 | Legionella_longbeachae_NSW150_uid46099 |
|  | 150 | Leptospira_biflexa_serovar_Patoc__Patoc_1__Ames__uid58511 |
|  | 151 | Leptospira_biflexa_serovar_Patoc__Patoc_1__Paris__uid58993 |
|  | 152 | Leuconostoc_gasicomitatum_LMG_18811_uid50385 |
|  | 153 | Leuconostoc_kimchii_IMSNU_11154_uid48589 |
|  | 154 | Listeria_innocua |
|  | 155 | Listeria_welshimeri_serovar_6b_SLCC5334 |
|  | 156 | Meiothermus_silvanus_DSM_9946_uid49485 |
|  | 157 | Methanobrevibacter_ruminantium_M1_uid45857 |
|  | 158 | Methanocaldococcus_FS406_22_uid42499 |
|  | 159 | Methanocaldococcus_infernus_ME_uid48803 |
|  | 160 | Methanococcus_vannielii_SB_uid58767 |
|  | 161 | Methanococcus_voltae_A3_uid49529 |
|  | 162 | Methanosarcina_acetivorans_C2A_uid57879 |
|  | 163 | Methanothermobacter_marburgensis_Marburg_uid51637 |
|  | 164 | Methylobacterium_chloromethanicum_CM4_uid58933 |
|  | 165 | Methylobacterium_populi_BJ001_uid58937 |
|  | 166 | Methylobacterium_radiotolerans_JCM_2831_uid58845 |
|  | 167 | Methylotenera_301_uid49469 |
|  | 168 | Mycobacterium_bovis_AF2122_97_uid57695 |
|  | 169 | Mycobacterium_bovis_BCG_Pasteur_1173P2_uid58781 |
|  | 170 | Mycobacterium_bovis_BCG_Tokyo_172_uid59281 |
|  | 171 | Mycobacterium_gilvum_PYR_GCK_uid59421 |
|  | 172 | Mycobacterium_KMS_uid58491 |
|  | 173 | Mycobacterium_marinum_M_uid59423 |
|  | 174 | Mycobacterium_MCS_uid58465 |
|  | 175 | Mycobacterium_tuberculosis_CDC1551_uid57775 |
|  | 176 | Mycobacterium_tuberculosis_F11_uid58417 |
|  | 177 | Mycobacterium_tuberculosis_H37Ra_uid58853 |
|  | 178 | Mycobacterium_tuberculosis_H37Rv_uid57777 |
|  | 179 | Mycobacterium_tuberculosis_KZN_1435_uid59069 |
|  | 180 | Mycoplasma_agalactiae_PG2 |
|  | 181 | Mycoplasma_agalactiae_uid46679 |
|  | 182 | Mycoplasma_capricolum_ATCC_27343_uid58525 |
|  | 183 | Mycoplasma_gallisepticum_R_low__uid57993 |
|  | 184 | Mycoplasma_hyorhinis_HUB_1_uid51695 |
|  | 185 | Mycoplasma_mobile_163K_uid58077 |
|  | 186 | Mycoplasma_penetrans_HF_2_uid57729 |
|  | 187 | Mycoplasma_pneumoniae_M129_uid57709 |
|  | 188 | Mycoplasma_synoviae_53_uid58061 |
|  | 189 | Neisseria_meningitidis_053442_uid58587 |
|  | 190 | Neisseria_meningitidis_alpha14 |
|  | 191 | Neisseria_meningitidis_FAM18_uid57825 |
|  | 192 | Neisseria_meningitidis_MC58_uid57817 |
|  | 193 | Neisseria_meningitidis_Z2491_uid57819 |
|  | 194 | Neorickettsia_sennetsu_Miyayama_uid57965 |
|  | 195 | Nitrobacter_winogradskyi_Nb_255_uid58295 |
|  | 196 | Nitrosococcus_halophilus_Nc4_uid46803 |
|  | 197 | Nitrosomonas_europaea_ATCC_19718_uid57647 |
|  | 198 | Nostoc_PCC_7120_uid57803 |
|  | 199 | Onion_yellows_phytoplasma_OY_M_uid58015 |
|  | 200 | Paenibacillus_polymyxa_E681_uid53477 |
|  | 201 | Paenibacillus_polymyxa_SC2_uid59583 |
|  | 202 | Pantoea_ananatis_LMG_20103_uid46807 |
|  | 203 | Pectobacterium_atrosepticum_SCRI1043_uid57957 |
|  | 204 | Pelobacter_propionicus_DSM_2379_uid58255 |
|  | 205 | Photorhabdus_asymbiotica_uid59243 |
|  | 206 | Polaromonas_naphthalenivorans_CJ2_uid58273 |
|  | 207 | Prevotella_ruminicola_23_uid47507 |
|  | 208 | Propionibacterium_acnes_KPA171202_uid58101 |
|  | 209 | Propionibacterium_acnes_SK137_uid48071 |
|  | 210 | Pseudoalteromonas_atlantica_T6c_uid58283 |
|  | 211 | Pseudomonas_entomophila_L48_uid58639 |
|  | 212 | Pseudomonas_fluorescens_Pf_5_uid57937 |
|  | 213 | Pseudomonas_fluorescens_Pf0_1_uid57591 |
|  | 214 | Pseudomonas_fluorescens_SBW25 |
|  | 215 | Pseudomonas_putida_F1_uid58355 |
|  | 216 | Pseudomonas_putida_GB_1_uid58735 |
|  | 217 | Pseudomonas_putida_KT2440_uid57843 |
|  | 218 | Pseudomonas_putida_W619_uid58651 |
|  | 219 | Pseudomonas_syringae_B728a_uid57931 |
|  | 220 | Psychrobacter_cryohalolentis_K5_uid58373 |
|  | 221 | Pyrobaculum_calidifontis_JCM_11548_uid58787 |
|  | 222 | Pyrobaculum_islandicum_DSM_4184_uid58635 |
|  | 223 | Pyrococcus_furiosus_DSM_3638_uid57873 |
|  | 224 | Ralstonia_eutropha_H16 |
|  | 225 | Ralstonia_pickettii_12D_uid58859 |
|  | 226 | Ralstonia_pickettii_12J_uid58737 |
|  | 227 | Rhizobium_leguminosarum_bv__trifolii_WSM1325_uid58991 |
|  | 228 | Rhizobium_leguminosarum_bv__trifolii_WSM2304_uid58997 |
|  | 229 | Rhizobium_leguminosarum_bv__viciae_3841_uid57955 |
|  | 230 | Rhodobacter_capsulatus_SB_1003_uid47509 |
|  | 231 | Rhodococcus_erythropolis_PR4_uid59019 |
|  | 232 | Rhodococcus_jostii_RHA1_uid58325 |
|  | 233 | Rhodospirillum_rubrum_ATCC_11170_uid57655 |
|  | 234 | Rickettsia_africae_ESF_5_uid58799 |
|  | 235 | Rickettsia_canadensis_McKiel_uid58159 |
|  | 236 | Rickettsia_conorii_Malish_7_uid57633 |
|  | 237 | Rickettsia_peacockii_Rustic_uid59301 |
|  | 238 | Rickettsia_prowazekii |
|  | 239 | Roseiflexus_RS_1_uid58523 |
|  | 240 | Rothia_dentocariosa_ATCC_17931_uid49331 |
|  | 241 | Ruegeria_TM1040_uid58193 |
|  | 242 | Salinispora_arenicola_CNS_205_uid58659 |
|  | 243 | Shewanella_ANA_3_uid58347 |
|  | 244 | Shewanella_denitrificans_OS217_uid58263 |
|  | 245 | Shewanella_frigidimarina_NCIMB_400_uid58265 |
|  | 246 | Shewanella_pealeana_ATCC_700345_uid58705 |
|  | 247 | Shewanella_piezotolerans_WP3_uid58745 |
|  | 248 | Shewanella_putrefaciens_CN_32_uid58267 |
|  | 249 | Shewanella_sediminis_HAW_EB3_uid58835 |
|  | 250 | Shewanella_woodyi_ATCC_51908_uid58721 |
|  | 251 | Shigella_dysenteriae_Sd197_uid58213 |
|  | 252 | Shigella_sonnei_Ss046_uid58217 |
|  | 253 | Sinorhizobium_medicae_WSM419_uid58549 |
|  | 254 | Spirochaeta_thermophila_DSM_6192_uid53037 |
|  | 255 | Staphylococcus_aureus_COL_uid57797 |
|  | 256 | Staphylococcus_aureus_ED98 |
|  | 257 | Staphylococcus_aureus_JH1_uid58457 |
|  | 258 | Staphylococcus_aureus_JH9_uid58455 |
|  | 259 | Staphylococcus_aureus_MRSA252_uid57839 |
|  | 260 | Staphylococcus_aureus_MSSA476_uid57841 |
|  | 261 | Staphylococcus_aureus_Mu3_uid58817 |
|  | 262 | Staphylococcus_aureus_Mu50_uid57835 |
|  | 263 | Staphylococcus_aureus_MW2_uid57903 |
|  | 264 | Staphylococcus_aureus_N315_uid57837 |
|  | 265 | Staphylococcus_aureus_NCTC_8325_uid57795 |
|  | 266 | Staphylococcus_aureus_Newman_uid58839 |
|  | 267 | Staphylococcus_aureus_RF122_uid57661 |
|  | 268 | Staphylococcus_aureus_USA300_FPR3757_uid58555 |
|  | 269 | Staphylococcus_aureus_USA300_TCH1516_uid58925 |
|  | 270 | Staphylococcus_epidermidis_ATCC_12228_uid57861 |
|  | 271 | Staphylococcus_epidermidis_RP62A_uid57663 |
|  | 272 | Staphylococcus_lugdunensis_HKU09_01_uid46233 |
|  | 273 | Staphylothermus_marinus_F1_uid58719 |
|  | 274 | Streptococcus_mutans_NN2025_uid46353 |
|  | 275 | Streptococcus_mutans_UA159_uid57947 |
|  | 276 | Streptococcus_pneumoniae_670_6B_uid52533 |
|  | 277 | Streptococcus_pneumoniae_70585_uid59125 |
|  | 278 | Streptococcus_pneumoniae_AP200_uid52453 |
|  | 279 | Streptococcus_pneumoniae_ATCC_700669_uid59287 |
|  | 280 | Streptococcus_pneumoniae_CGSP14_uid59181 |
|  | 281 | Streptococcus_pneumoniae_D39_uid58581 |
|  | 282 | Streptococcus_pneumoniae_G54_uid59167 |
|  | 283 | Streptococcus_pneumoniae_Hungary19A_6_uid59117 |
|  | 284 | Streptococcus_pneumoniae_JJA_uid59121 |
|  | 285 | Streptococcus_pneumoniae_P1031_uid59123 |
|  | 286 | Streptococcus_pneumoniae_R6_uid57859 |
|  | 287 | Streptococcus_pneumoniae_Taiwan19F_14_uid59119 |
|  | 288 | Streptococcus_pneumoniae_TCH8431_19A_uid49735 |
|  | 289 | Streptococcus_pneumoniae_TIGR4_uid57857 |
|  | 290 | Streptococcus_pyogenes_M1_GAS_uid57845 |
|  | 291 | Streptococcus_pyogenes_Manfredo_uid57847 |
|  | 292 | Streptococcus_pyogenes_MGAS10270_uid58571 |
|  | 293 | Streptococcus_pyogenes_MGAS10394_uid58105 |
|  | 294 | Streptococcus_pyogenes_MGAS10750_uid58575 |
|  | 295 | Streptococcus_pyogenes_MGAS2096_uid58573 |
|  | 296 | Streptococcus_pyogenes_MGAS315_uid57911 |
|  | 297 | Streptococcus_pyogenes_MGAS5005_uid58337 |
|  | 298 | Streptococcus_pyogenes_MGAS6180_uid58335 |
|  | 299 | Streptococcus_pyogenes_MGAS8232_uid57871 |
|  | 300 | Streptococcus_pyogenes_MGAS9429_uid58569 |
|  | 301 | Streptococcus_pyogenes_NZ131_uid59035 |
|  | 302 | Streptococcus_pyogenes_SSI_1_uid57895 |
|  | 303 | Streptococcus_sanguinis_SK36_uid58381 |
|  | 304 | Streptococcus_suis_05ZYH33_uid58663 |
|  | 305 | Streptococcus_suis_98HAH33_uid58665 |
|  | 306 | Streptococcus_suis_BM407_uid59321 |
|  | 307 | Streptococcus_suis_P1_7 |
|  | 308 | Streptococcus_suis_SC84_uid59323 |
|  | 309 | Streptococcus_thermophilus_CNRZ1066_uid58221 |
|  | 310 | Streptococcus_thermophilus_LMD_9_uid58327 |
|  | 311 | Streptococcus_thermophilus_LMG_18311_uid58219 |
|  | 312 | Streptomyces_avermitilis_MA_4680_uid57739 |
|  | 313 | Streptomyces_scabiei_87_22_uid46531 |
|  | 314 | Sulfolobus_acidocaldarius_DSM_639_uid58379 |
|  | 315 | Sulfolobus_tokodaii_7_uid57807 |
|  | 316 | Sulfurihydrogenibium_azorense_Az_Fu1_uid58121 |
|  | 317 | Sulfurimonas_denitrificans_DSM_1251_uid58185 |
|  | 318 | Synechococcus_CC9902_uid58323 |
|  | 319 | Synechococcus_elongatus_PCC_6301_uid58235 |
|  | 320 | Synechococcus_elongatus_PCC_7942_uid58045 |
|  | 321 | Synechococcus_JA_2_3B_a_2_13__uid58537 |
|  | 322 | Synechococcus_JA_3_3Ab_uid58535 |
|  | 323 | Synechococcus_PCC_7002_uid59137 |
|  | 324 | Thermoanaerobacter_italicus_Ab9_uid46241 |
|  | 325 | Thermoanaerobacter_pseudethanolicus_ATCC_33223_uid58339 |
|  | 326 | Thermococcus_gammatolerans_EJ3_uid59389 |
|  | 327 | Thermococcus_kodakarensis_KOD1_uid58225 |
|  | 328 | Thermoplasma_volcanium_GSS1_uid57751 |
|  | 329 | Thermosipho_africanus_TCF52B_uid59095 |
|  | 330 | Thermotoga_lettingae_TMO_uid58419 |
|  | 331 | Thermotoga_maritima_MSB8_uid57723 |
|  | 332 | Thermotoga_naphthophila_RKU_10 |
|  | 333 | Thioalkalivibrio_HL_EbGR7_uid59179 |
|  | 334 | Treponema_denticola_ATCC_35405_uid57583 |
|  | 335 | uncultured_Termite_group_1_bacterium_phylotype_Rs_D17 |
|  | 336 | Ureaplasma_urealyticum_serovar_10_ATCC_33699_uid59011 |
|  | 337 | Vibrio_cholerae_M66_2_uid59355 |
|  | 338 | Vibrio_cholerae_MJ_1236_uid59387 |
|  | 339 | Vibrio_cholerae_O1_biovar_El_Tor_N16961_uid57623 |
|  | 340 | Vibrio_cholerae_O395_uid58425 |
|  | 341 | Vibrio_Ex25 |
|  | 342 | Vibrio_vulnificus_CMCP6 |
|  | 343 | Vibrio_vulnificus_YJ016_uid58007 |
|  | 344 | Wolbachia_endosymbiont_of_Drosophila_melanogaster_uid57851 |
|  | 345 | Wolbachia_endosymbiont_TRS_of_Brugia_malayi_uid58107 |
|  | 346 | Xanthomonas_albilineans |
|  | 347 | Xanthomonas_campestris_8004_uid57595 |
|  | 348 | Xanthomonas_campestris_ATCC_33913_uid57887 |
|  | 349 | Xanthomonas_campestris_B100 |
|  | 350 | Xenorhabdus_bovienii_SS_2004_uid46345 |
|  | 351 | Yersinia_pseudotuberculosis_IP_31758_uid58487 |
|  | 352 | Yersinia_pseudotuberculosis_IP_32953_uid58157 |
|  | 353 | Yersinia_pseudotuberculosis_PB1__uid59153 |
|  | 354 | Yersinia_pseudotuberculosis_YPIII_uid59151 |

We downloaded 1212 fully sequenced bacterial genomes from the NCBI FTP site (ftp://ftp.ncbi.nih.gov/genomes/Bacteria/) on 14 Dec 2010. Using the NCBI taxonomy, 390 genomes were removed to guarantee that every genus has at least two genomes. The remaining 822 genomes were assigned to training and test groups. Genomes from a species were assigned to one and only one set, either the training set or the test set. In the end, we generated 468 training genomes and 354 test genomes.
