# Supplementary material for: MetaBinG: Using GPUs to Accelerate Metagenomic Sequence Classification
Source: PLoS One. 2011 Nov 23;6(11):e25353. doi: 10.1371/journal.pone.0025353 (PMC3223155; doi:10.1371/journal.pone.0025353)
Supplement: Table S2 — Accuracy of Phymm at different ranks. The accuracy of Phymm was reported at different ranks. The data sets, the software and parameters are all the same as in Table 2. (DOC) [file pone.0025353.s002.doc]

**Table S2. Accuracy of Phymm at different ranks.**

|  | Phylum | Class | Order | Family | Genus |
| --- | --- | --- | --- | --- | --- |
| 100 | 53.62% | 42.94% | 34.41% | 28.76% | 26.33% |
| 200 | 64.21% | 54.38% | 47.23% | 40.56% | 38.64% |
| 300 | 70.71% | 60.99% | 54.60% | 47.88% | 45.56% |
| 400 | 73.36% | 65.11% | 59.10% | 52.18% | 49.58% |
| 500 | 76.02% | 68.22% | 63.11% | 56.24% | 53.64% |
| 600 | 78.47% | 71.21% | 66.10% | 59.29% | 56.64% |
| 700 | 79.89% | 72.18% | 67.94% | 60.99% | 58.47% |
| 800 | 81.86% | 74.69% | 70.25% | 63.11% | 60.14% |
| 900 | 82.40% | 75.68% | 71.47% | 64.49% | 61.69% |
| 1000 | 84.18% | 77.68% | 73.70% | 66.67% | 63.98% |

The accuracy of Phymm was reported at different ranks. The data sets, the software and parameters are all the same same in Table 1.
